# Deciphering the functional role of insular cortex stratification in trigeminal neuropathic pain

**DOI:** 10.1186/s10194-024-01784-5

**Published:** 2024-05-10

**Authors:** Jaisan Islam, Md Taufiqur Rahman, Elina KC, Young Seok Park

**Affiliations:** 1 Department of Medical Neuroscience, College of Medicine, Chungbuk National University, Cheongju, Korea; 2 Department of Neurosurgery, Chungbuk National University Hospital, Cheongju, Korea; 3 Department of Neuroscience and Regenerative Medicine, Medical College of Georgia, Augusta University, Augusta, GA USA

**Keywords:** Trigeminal neuropathic pain, Insular cortex, Neuroplasticity, Neuromodulation, Therapeutic potential of insular cortex

## Abstract

Trigeminal neuropathic pain (TNP) is a major concern in both dentistry and medicine. The progression from normal to chronic TNP through activation of the insular cortex (IC) is thought to involve several neuroplastic changes in multiple brain regions, resulting in distorted pain perception and associated comorbidities. While the functional changes in the insula are recognized contributors to TNP, the intricate mechanisms underlying the involvement of the insula in TNP processing remain subjects of ongoing investigation. Here, we have overviewed the most recent advancements regarding the functional role of IC in regulating TNP alongside insights into the IC’s connectivity with other brain regions implicated in trigeminal pain pathways. In addition, the review examines diverse modulation strategies that target the different parts of the IC, thereby suggesting novel diagnostic and therapeutic management of chronic TNP in the future.

## Background

Trigeminal neuropathic pain (TNP) represents a significant medical challenge, characterized by episodic, intense pain affecting the trigeminal nerve, crucial for facial and head sensation. Triggered by routine activities such as eating, speaking, and facial cleansing, TNP manifests in highly sensitive areas, often unrelated to the actual pain sites. Its etiology includes trigeminal nerve root compression, demyelinating diseases, and alterations in pain-related central neural circuits, leading to abnormal neural firing [9, 22, 50]. Current treatment modalities for TNP encompass pharmacological interventions, surgical procedures, and alternative therapies. Primary pharmacological treatments include sodium channel blockers such as carbamazepine and oxcarbazepine, supplemented by tricyclic antidepressants and anticonvulsants [37]. The diagnosis of TNP is challenging due to its rarity and potential misidentification as dental issues, migraines, or temporomandibular joint disorders, complicating effective management [63, 91]. While local anesthetic or steroid injections offer transient relief, invasive surgical interventions pose risks, including further nerve damage, highlighting the need for advancements in neuromodulation techniques [45].

The insular cortex (IC), deeply situated within the lateral sulcus of the brain’s cerebral cortex, plays a pivotal role in emotion, cognition, and pain perception. Research has established its critical involvement in the TNP processing pathway, evidenced by observed alterations of gray matter volume (GMV) and neurotransmitters in TNP patients and animal models [50, 61, 68, 100, 101]. However, the role and mechanism of IC in TNP and its management, compared to other neuropathies, have yet to be fully explored. The IC integrates trigeminal nociceptive inputs from various brain regions, making it a target for modulating trigeminal pain through lesioning, neurostimulation, and pharmacological strategies [3, 50, 78, 98]. Therefore, given the growing emphasis on the IC in TNP management research, further investigation into its therapeutic possibilities for TNP is paramount. This review aims to elucidate the IC’s significance in TNP and evaluate the potential of various modulation approaches, alongside discussing current and future diagnostic and therapeutic strategies for chronic TNP management involving the IC.

## Methods

### Procedures of literature search and study selection

In this comprehensive review, we conducted a systematic literature search using PubMed and the Scopus Index as primary resources to explore the modulation of the IC in the context of TNP. Our search strategy involved a computerized examination of journal articles without restricting the publication date, employing a broad spectrum of keywords such as “pain,” “trigeminal neuropathic pain,” “insular cortex,” “orofacial pain,” and “trigeminal neuralgia” to ensure comprehensive coverage of the topic.

To thoroughly investigate the functional role of the IC in TNP, our review included studies encompassing both animal models and human subjects. The inclusion of animal studies allowed to understand foundational biological processes and experimental therapeutics, while human studies provided insights into clinical manifestations, imaging findings, and therapeutic outcomes. This dual approach enabled a holistic understanding of the IC’s role across different experimental and clinical settings.

We selected 164 non-duplicated entries, employing a rigorous criterion that focused on the relevance to our area of interest, and the novelty of findings related to the role of the IC in TNP. An initial screening of titles and abstracts, primarily conducted by the first author, identified 107 studies that significantly contributed to our understanding of the IC’s involvement in TNP. These articles were chosen based on their discussions about alterations to the IC following the condition and the various neuromodulation techniques targeting the IC. Our review synthesizes findings associated with the IC in the context of TNP, which encompasses orofacial pain, trigeminal neuropathic pain, and trigeminal neuralgia, ensuring a scientifically robust selection of literature.

### Structure of insular cortex

The IC, integral to the processing of multimodal inputs, is delineated by its anatomical location and cytoarchitecture across species. IC is divided into two parts: AIC and PIC. The AIC is comprised of three short gyri which are involved in processing emotions, empathy and social awareness. On the other hand, PIC is comprised of two long gyri which are implicated in perception, motor control, self-awareness and sensory integration [34, 48, 92]. In rats, it lies over the claustrum, bordered rostrodorsally by the lateral frontal and primary somatosensory cortices (SI), caudodorsally by the secondary somatosensory cortex (SII), ventrally by the piriform cortex, and caudally by the perirhinal cortex [60, 86]. In primates, it resides within the lateral sulcus’s fold, comprising anterior and posterior sections with distinct connectivity profiles [96]. Cytoarchitecturally, the IC in primates, including humans, is divided into granular, dysgranular, and agranular areas. The agranular insular cortex (aIC), situated in the AIC, is characterized by its prominent layers II–III, V, and VI, and is mainly associated with efferent functions. The dysgranular insular cortex (dIC), found between the granular and agranular areas, has fewer granule cells in layer IV and a significant layer V. It plays a role in integrating sensory and emotional information, with both afferent and efferent functions. The granular insular cortex (gIC), primarily located in the PIC, is known for its pronounced layer IV and is more associated with afferent pathways [3, 33, 61].

### Functions of insular cortex

Functionally, the insula is implicated in diverse processes. It acts as the primary gustatory cortex [14], visceral [88], and thermosensory cortex [20], embodying the primary interoceptive cortex that reflects the body’s physiological and homeostatic conditions [61]. The PIC, specifically, is known to receive substantial sensory input from cortical sources, highlighting IC’s integral role in comprehensively processing somatosensory information [38]. This extensive sensory integration indicates the capability of IC to mediate complex interactions between different sensory modalities and the neural network involved in higher-order processing. Its role also extends to embodying consciousness and self-recognition, evidenced by activation upon viewing one’s images [28] during awareness of heartbeat, bodily control, and emotions [19]. Its integration with the limbic system underlines its crucial role in emotional processing, including negative behaviors like fear and anxiety [32, 52] and positive emotions such as happiness [61].

Cognitively, the IC is involved in aversive and affective learning, as shown in rat studies [53, 85], aligning with its role in the salience network [94]. It participates in anticipating future states, prediction error computation, and risk estimation, responding predictively to relevant physiological stimuli [6, 40, 67]. This mediation between physiological states and motivated behaviors underscores its significance in both normal and pathological conditions [6, 21, 79].

Pathologically, variations in insular function and structure are linked to anxiety disorders [82], major depression [5, 76], autism spectrum disorders [95], schizophrenia, obesity, and addiction [30], highlighting its pivotal role across a spectrum of mental health and behavioral conditions. In addition, the IC plays a multifaceted role in motor control by integrating sensory, emotional, and cognitive information to influence motor functions, including planning, execution, learning, and adaptation. It coordinates with other motor control areas, processes pain, and modulates autonomic responses, highlighting its integral role in the complex interplay between motor activities and internal states [87].

### Chemoarchitectural features of IC involved in trigeminal pain processing

The IC significantly influence TNP through its complex chemoarchitectural characteristics. The extensive expression of neurotransmitter receptors situated in the IC, including opioid, cannabinoid, dopaminergic, and glutamate receptors, enzymatic activities, and specific neurocytological profiles underpins its critical function in the TNP [39, 62]. aIC and dIC, known for their reduced myelination and distinct acetylcholinesterase activity, suggest a unique substrate that may influence signal propagation speed and integration, particularly relevant to the processing of TNP signals [36]. The presence of µ-, δ-, and κ-opioid, along with nicotinic acetylcholine receptors in the IC, highlights its role in modulating pain relief, reward, and addiction. In addition, cannabinoid receptors and serotonergic receptors such as 5-HT1A, 5-HT2A, 5-HT2C, 5-HT3, and potentially 5-HT4, 5, 6, 7, within the IC influence the perception of TNP [62, 93].

In TNP, alteration in dopaminergic neurotransmission circuitry affects multiple brain regions such as IC and nucleus accumbens core (NAcc). Since IC has output projections towards NAcc and NAcc modulation have been found to be involved in TNP, IC dopaminergic neurotransmission can influence TNP [15, 38, 39, 49].

Studies have further elucidated that glutamatergic mechanisms within the IC contribute to central sensitization and the modulation of TNP. Alterations in glutamate receptor expression, such as NMDAR and AMPAR, have been associated with TNP, suggesting the pivotal role of excitatory neurotransmission and synaptic plasticity within the IC [50, 59]. GABAergic mechanisms within the IC have also been increasingly recognized for their contribution to neuroplasticity and neuromodulation, particularly in the context of emotional regulation and interoceptive awareness [39].

In addition, increased Phospho-Extracellular Signal-Regulated Kinase (pERK) activation in the IC is associated with central sensitization of TNP [3, 98].

### Relationship between insular cortex activity and trigeminal neuropathic pain in human studies

Recent evidence demonstrated the critical involvement of both AIC and PIC in the TNP processing pathway, with alterations in GMV commonly observed in TNP cases [26, 27, 42, 47, 64, 72]. Functional imaging techniques, such as Positron Emission Tomography (PET) and functional Magnetic Resonance Imaging (fMRI), have confirmed the activation of IC in response to nociceptive orofacial stimuli, with the AIC implicated in higher-level pain interpretation and the PIC in basic sensory pain processing [10, 11, 31, 71, 89]. Furthermore, individuals with trigeminal neuralgia (TN) showed significant functional connectivity changes and microstructural integrity alterations in the white matter volume (WMV) of the IC [100, 107]. The role of IC extends beyond processing orofacial sensations to integrating sensory inputs from both primary (SI) and secondary (SII) somatosensory cortices [57]. In the IC, there is a distinct pattern of intra-insular outputs, with a greater number from the PIC than the AIC, suggesting a directional, caudal-to-rostral flow of information within the IC [39]. Therefore, interruptions in connectivity between the AIC and PIC, whether due to lesions or neuromodulation, have been associated with impairments in trigeminal pain and temperature sensations [31].

The AIC not only processes pain intensity and the emotional dimensions of pain experiences but also plays a crucial role in anticipating pain and facilitating human awareness [7, 31]. Intriguingly, after effective treatment for TN, the ventral AIC often shows a normalization of GMV and cortical thickness, suggesting neuroplastic adjustments that correlate with clinical improvement [26, 27].

On the other hand, PIC is essential for processing pain and tactile sensations, serving as a primary cortical hub for integrating internal and external bodily signals [10, 38]. Its role in somesthesis is underlined by its connections with the spinothalamic tract and the reception of nociceptive and thermoceptive information via the lamina-I-spinothalamocortical pathway from the posterior thalamic nuclei, which are crucial for the sensory discriminative aspects of trigeminal pain [71, 83].

### Relationship between insular cortex activity and trigeminal neuropathic pain in preclinical studies

Recent preclinical studies have been pivotal in elucidating the insula’s role in trigeminal pain perception, building on the findings from human research. Rodent and monkey studies confirm the IC’s response to trigeminal pain through its activation to oralfacial nociceptive stimulation [3, 57, 71, 89]. Furthermore, animal studies reveal that IC lesions can mitigate neuropathic and inflammatory pain [44].

At the molecular level, trigeminal nerve injuries activate the ERK-CREB pathway in the IC, leading to an upregulation of glutamate receptors (AMPA and NMDA) and a downregulation of inhibitory potassium channels activity, which promotes neuronal long-term potentiation associated with trigeminal pain [98]. Enhanced excitatory neural responses in the dorsal IC following sensory stimulation have been observed in rats with trigeminal neuropathy [35]. Furthermore, plastic changes in neuronal circuitries from the IC to the trigeminal nucleus caudalis (TNC) may amplify responses to peripheral noxious stimulation [78].

The AIC is significantly responsive to alterations in nociceptive inputs from trigeminal afferents. Enhanced phosphorylation of ERK-1/2 in layers II-III, areas known for housing nociceptive-specific neurons, indicates the active involvement of AIC in TNP processing [3]. Additionally, the AIC influences spinal cord activities through top-down modulation, likely mediated by noradrenergic outputs from the locus coeruleus (LC), which interacts with inhibitory inputs from the lateral parabrachial nucleus (PBN) and raphe magnus nucleus (RMN). This complex interplay indicates the integral role of AIC in both initiating and modulating the pain response, particularly in conditions like TN where neurovascular compression is a significant factor [16, 56].

On the contrary, the PIC plays a critical role in TNP by regulating orofacial sensory-motor functions, and serving as a hub for thalamic sensory inputs [2, 81, 103]. Research in monkeys shows the PIC receives specific thermal and pain signals, integrating them with broader autonomic and limbic systems [20]. Additionally, the PIC processes a diverse array of information, including sensory discrimination, highlighted by increased brain activity in conditions like brush-evoked allodynia. This demonstrates its capacity for neuroplasticity and neuromodulation, as evidenced by studies on somatosensory-evoked potentials [10, 23].

Further, the PIC integrates sensory, autonomic, motor, associative, and limbic inputs, maintaining strong internal connections that facilitate its involvement in varied stimuli and emotional states [38, 84]. In the chronic constriction injury of the Infraorbital neve (CCI-ION) rat model for TNP, increased activity of dysgranular PIC (dPIC) glutamatergic neurons (dPICg) in response to the nerve injury had been observed, which was associated with enhanced expression of pERK and CREB in the dPIC. This suggests the nociceptive processing role of dPIC involving glutamatergic neural networks during TNP [50].

**Table 1:** Overview of clinical and preclinical studies on the insula in trigeminal neuropathic pain

Subject condition	Stimulation method	Analyzing procedure	Brain region	Findings	Reference
TNP	Innocuous brushing of the lip	VBM	Ipsilateral AIC	Reduced GMV	[42]
TNP	Innocuous brushing of the lip	VBM	Contralateral PIC	Increased GM	[42]
TN	Light tactile stimulation	VBM, CTA	Ipsilateral AIC and PIC	Decreased cortical thickness	[26]
TN	Light tactile stimulation	VBM	Ipsilateral IC	Reduced GMV	[105]
TN	Daily activities (talking, chewing)	DWI, SIFT, SVM	Bilateral IC	Altered WMV connectivity and microstructural integrity	[107]
TNP	Daily activities (talking, chewing)	fMRI	Ipsilateral AIC, bilateral PIC	Decreased cortical thickness	[47]
TN	Electrical stimulation, tapping or lightly touching the skin	VBM	IC	Reduced GMV	[73]
TN	Daily activities	VBM, CTA	Right Ventral AIC and bilateral PIC	Reduced GMV by 9% and 10.4%	[27].
TNP	Mechanical (brush) and thermal (cold and heat)	fMRI	AIC	Increased activity	[8]
Healthy	Gaseous CO2 trigeminal stimuli	fMRI	Operculo-Insula	Increased activity	[68]
TN	Classical precipitating factors (speaking, swallowing, shaving, yawning, etc.)	CTA, 3D vertex-wise analysis	Left IC	Reduced local gyrification index and decreased cortical thickness	[100]
TN	Classical precipitating factors (speaking, swallowing, shaving, yawning, etc.)	fMRI	Contralateral AIC	Increased activity	[72]
TN	Thermal and pulsed radiofrequency	fMRI	Both AIC and PIC	Enhanced BOLD signal	[1]
TN	Classical precipitating factors (speaking, swallowing, shaving, yawning, etc.)	VBM	IC	Reduced GMV	[99]
CCI-ION rat	Light, moving strokes on the infraorbital skin	IFC	AIC (Dysgranular, agranular)	Increased ERK phosphorylation	[3]
CCI-ION rat	Electrical and mechanical noxious stimulation	IFC, WB, electrophysiology	IC (Granular, dysgranular)	Increased pERK-CREB pathway, decreased neural activity	[98]
Pl-ION rat	Electrical stimulation of mandibular molar pulp	IFC	Insular oral region	Enhanced neural activity	[80]
Naïve rat	Mechanical stimulation with wire	High-resolution epicortical evoked potential mapping	AIC	Decreased activity	[10]
Naïve monkey	Noxiuos cold stimuli	fMRI	Dorsal PIC	Increased activity	[18]
Chronic corneal pain mice	Chemical corneal sensitivity test	fMRI	IC	Upregulated amplitude of low-frequency fluctuation (ALFF)	[103].
Adult male C57BL/6 mice	In vitro chemical (α-CGRP and CGRP8–37) stimulation	Whole-cell patch-clamp recording	IC	Enhanced presynaptic glutamate release	[66]
adult vesicular GABA transporter (VGAT)-Venus line A transgenic rats	In vitro injection of depolarizing and hyperpolarizing current pulses	Multiple whole-cell patch-clamp recordings	IC	Increased excitatory presynaptic currents (pEPSC) amplitudes	[77]
CCI-ION rat	Mechanical and thermal stimulation	IFC	dPIC	Increased glutamatergic neural activity and enhanced CREB and pERK expression	[50]
Trigeminal nerve transection rat	Electrical stimulation	In vivo optical imaging, whole-cell patch-clamp recording	Layer II/III dorsal IC	Increased neural activity	[35]

TN = trigeminal neuralgia, TNP = trigeminal neuropathic pain, fMRI = functional magnetic resonance imaging, AIC = anterior insular cortex, PIC = posterior insular cortex, WMV = white matter volume, GMV = gray matter volume, BOLD = blood oxygen level-dependent. CCI-ION = chronic constriction injury of the infraorbital nerve, IFC = immunofluorescence, WB = western blot, RAIC = rostral agranular insular cortex, dPIC = dysgranular posterior insular cortex, DWI = diffusion-weighted magnetic resonance imaging, SVM = support vector machine classification, VBM = voxel-based morphometry, CTA = cortical thickness analysis

### Projections of insular cortex to and from other trigeminal neuropathic pain-associated brain regions

**Fig. 1:**
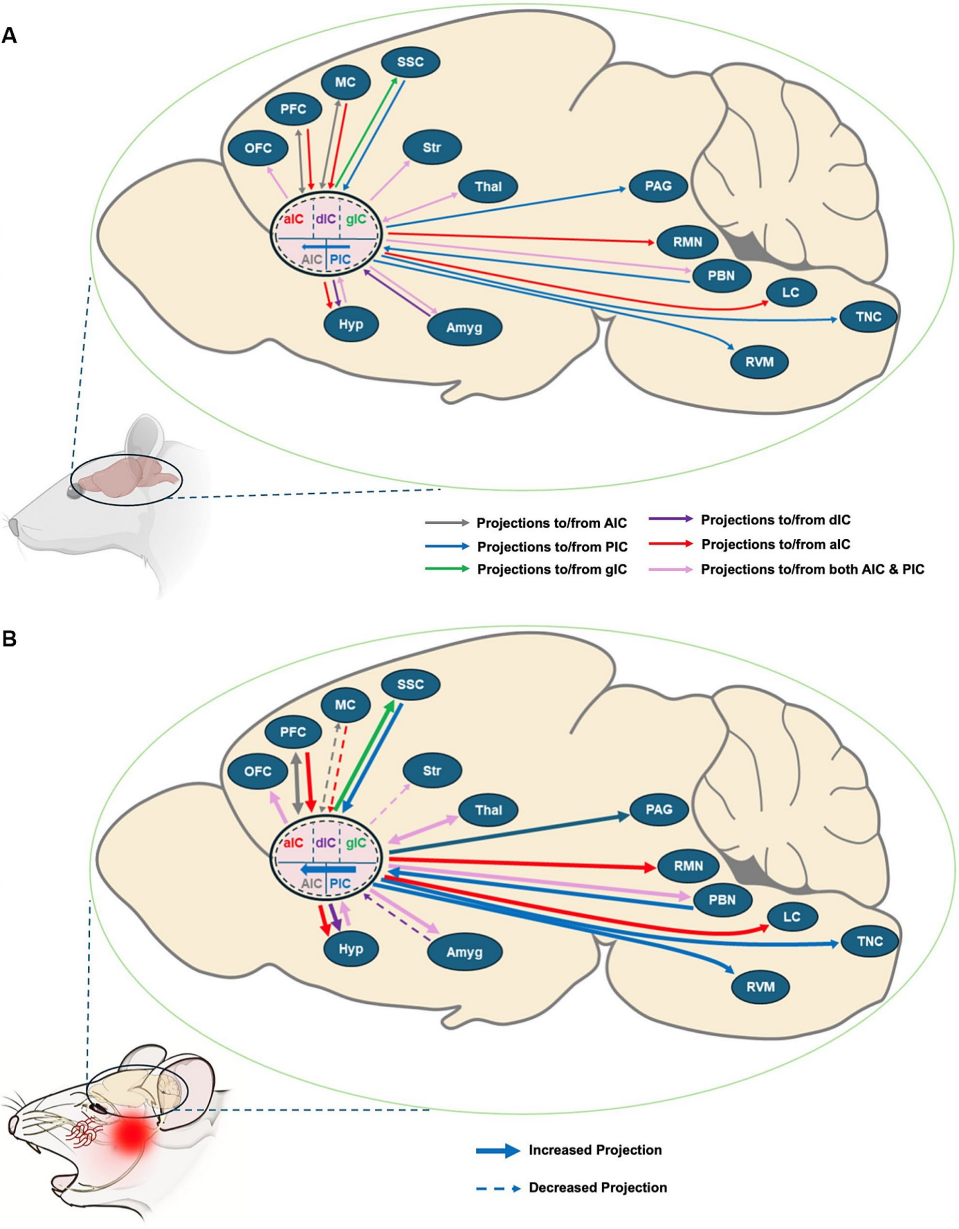
Connections of IC with different brain regions. **(A)** Projections of IC with other brain regions. **(B)** Altered projections of IC with other brain regions in TNP. IC = insular cortex, OFC = orbitofrontal cortex, PFC = prefrontal cortex, MC = motor cortex, SSC = somatosensory cortex, NAc = nucleus accumbens, Str = striatum, Thal = thalamus, Hyp = hypothalamus, Amyg = amygdala, PAG = periaqueductal gray, RMN = raphe magnus nucleus, PBN = parabrachial nucleus, LC = locus coeruleas, RVM = rostral ventromedial medulla, TNC = trigeminal nucleus caudalis

The IC is a hub for processing multisensory information, with each of its subdivisions playing distinct roles in handling various types of sensory data. Beyond receiving inputs from several brain areas, the IC has excitatory projections to different brain structures as well, enriching its multisensory processing capabilities with emotional and affective context (Fig. 1). This convergence ensures that sensory information processed within the IC is seamlessly integrated with limbic information, highlighting its comprehensive role in sensory perception and emotional regulation. However, it is important to note that most of the studies documenting these functions and projections are based on animal models, which could have implications for direct translational relevance to human anatomy and pathology.

### Trigeminal nucleus caudalis

In TNP research, significant insights have been gained into the functional anatomy of the IC and its connections to the TNC and the trigeminal subnucleus oralis (Vo) [3, 98]. Descending projections from the IC, specifically targeting lamina I of the medullary dorsal horn and Vo, underscore the IC’s pivotal role in conveying orofacial nociceptive information [2, 20, 104]. The gIC and dIC have been shown to project to both the rostral and caudal parts of TNC laminae I/II, as well as to Vo, through the corticotrigeminal pathway [2, 25, 78, 89, 98, 104]. In addition, inhibition of dPICg activity demonstrated reduced TNC activity in CCI-ION rats highlighting a direct modulatory control of IC over TNC [50].

### PAG and RVM

The gIC and the dIC have been identified as significant contributors to the endogenous descending pain-modulatory system, projecting into key structures such as the periaqueductal gray (PAG) and the rostral ventromedial medulla (RVM). These projections regulates orofacial pain processing in neurons within laminae I/II of the TNC and the Vo. The caudal regions of gIC/dIC predominantly project to the lateral PAG, with both caudal and rostral regions showing fewer projections to the ventrolateral PAG. Additionally, axonal fibers from both rostral and caudal gIC/dIC regions extend and terminate in the RVM, further highlighting IC’s indirect connection to TNC via the PAGl and RVM [77, 89].

### PBN and KF

The dIC and gIC have robust connections to the parabrachial nucleus (PBN) and the Kölliker-Fuse nucleus (KF), both of which, in turn, directly project to the TNC and the Vo [89]. PBN also projects ascending output to these subregions, a commonality across all mammals, mediated through the ventromedial basal nucleus (VMb) [20]. In addition, the aIC is known to send glutamatergic excitatory outputs to the lateral PBN [56]. These connections facilitate the integration of sensory and nociceptive information in the IC.

### Locus coeruleus

The aIC selectively projects to the inhibitory neurons in the locus coeruleus (LC) and activates inhibitory descending pain projection pathways [51, 77]. This is further supported by evidence showing that the rostral part of the aIC sends glutamatergic excitatory outputs to the LC [56], indicating a complex interplay of excitatory inputs from the insula to the LC. It has been shown that noradrenaline from the LC can enhance TNP [29].

### Raphe magnus nucleus

The rostral part of the aIC modulates pain through excitatory glutamatergic outputs to the inhibitory neurons within the raphe magnus nucleus (RMN), a key component of the brain’s pain management system [56]; Nakaya et al. [77]. This pathway between the aIC and RMN significantly contributes to the descending modulatory systems that regulate nociception, illustrating the essential dynamic balance of excitatory and inhibitory interactions within the brain’s endogenous pain management mechanisms [93].

### Amygdala

Within the IC, a significant expression of the 5-HT1A receptor is observed in projection neurons targeting specific sub-nuclei of the amygdala, namely the central or basolateral nuclei, with approximately 75–80% of these insula-amygdala projection neurons containing 5-HT1A [52]. The amygdala plays a crucial role in the emotional dimensions of trigeminal pain perception by receiving projections from various IC nuclei. These connections are essential for integrating emotional and nociceptive signals in TNP conditions [4, 97].

The AIC and PIC are connected to the amygdala’s central, lateral, dorsolateral, and basolateral nuclei, demonstrating a comprehensive network of insular projections that influence the diverse neural functions of the amygdala [90]. The PIC is noted for its strong subcortical outputs primarily directed towards the central nucleus of the amygdala (CeA) [38, 57]. In contrast, the AIC exhibits limited glutamatergic projections to the amygdala, with only sparse projections observed to the extended amygdala [38, 39, 56]. In patients with classical TN, enhanced functional connectivity has been observed between IC and amygdala [99].

In terms of projections from the amygdala, dIC receives dense projections from various amygdaloid nuclei, including the lateral, basolateral, and central nuclei. These amygdaloid inputs are distributed across all layers of the dIC [56].

### Striatum

Both the AIC and PIC have significant projections to the NAcc and caudate putamen (CPu), reflecting distinct interaction patterns with the striatum by different insular subdivisions. In contrast, no feedback connections from the striatum to the IC have been identified, emphasizing a one-way flow of information from the IC to the striatum, underlying the complex communication involved in the processing of emotional and sensory information [38, 39]. TNP patients showed a decrease in GMV in the NAcc and CPu [42, 46]. The striatum plays a crucial role in trigeminal pain perception and modulation by integrating information from the nociceptive spinothalamic tract and descending cortical pathways. Its involvement in dopamine signaling indicates its significant impact on orofacial pain perception [65]. Moreover, NAcc has been found to influence TNP through its GABAergic medium spiny neurons and their projections to pain-regulating pathways [49]. While this could hypothetically suggest changes in the projections from the IC to these areas of the striatum, further investigations are required to directly establish this implication.

### Thalamus

The aIC is integrally connected to the thalamus, receiving a diverse range of inputs from the mediodorsal and centro-median nuclei for broad sensory integration [39, 90]. It also receives specific projections from the thalamus’s sub-medius and central lateral nuclei, as well as the parvicellular part of the ventral posterior nucleus [56]. Whereas, the gIC and dIC subregions form deep cortical layers reciprocal projections with the thalamus by receiving extensive sensory inputs and sending significant outputs back, particularly through the VPM, VPMpc, and VPL nuclei, which handle somatosensory, gustatory, and visceral signals [38, 39, 90]. Specifically, the dIC is targeted by nociceptive pathways from the ventral medial thalamic nucleus, highlighting a specific pathway for pain signals [7]. In TN patients, functional connectivity from the left IC to the thalamus was found to be increased [100]. These connections indicate the intricate relationship of IC with the thalamus.

### Hypothalamus

Neurons within the IC project to both the rostral and caudal sections of the lateral hypothalamus (LH), with approximately 75% of these insula-LH projection neurons expressing the 5-HT1A receptor [52]. Neurons from both the aIC and the dIC extend their axons to the LH area [57]. The dIC, in particular, maintains reciprocal connections with the LH [56]. The projections from the aIC to the hypothalamus and brainstem are posited to play a critical role in the modulation of descending pain inhibitory control [93].

### Somatosensory cortex I/II

The somatosensory cortex I (SI) is primarily influenced by dominant afferent projections from the gIC, forming a potential “spinal–gIC–SI–spinal” positive feedback loop that could underpin the persistence of allodynia [11]. The critical involvement of IC in the somatosensory neural network is indicated by its extensive sensory input from primary and secondary cortical regions, especially targeting the PIC’s excitatory neurons. On the contrary, optical imaging has revealed significant excitatory propagation from the gIC to both SI and SII [35, 39, 56]. This complex network of projections and feedback loops involving the IC, SI, and SII highlights the intricate mechanisms through which the brain processes and modulates somatosensory and nociceptive signals.

### Motor cortex

The motor cortex and AIC has reciprocal connections between them, whereas, the aIC predominantly receives inputs from the motor cortex [39, 75].

### Prefrontal cortex

Connectivity from prefrontal cortex (PFC) regions to the AIC is notably prominent, particularly targeting inhibitory neurons within the aIC. Further supporting this intricate connectivity, studies have revealed strong direct intracortical pathways linking the IC with the medial PFC (mPFC) [2, 54]. In contrast, AIC exhibits a bidirectional strong connection with the ventrolateral PFC, highlighting the nuanced interplay between different cortical areas in processing and integrating a wide range of cognitive functions [31].

### Orbitofrontal cortex

IC has excitatory projections to the OFC and in TNP, this projections get enhanced due to increased excitatory inputs from layer IV to layer II/III pyramidal neurons in the insular-orbitofrontal region [35, 70].

### Periodontal ligament and dental pulp

The dIC and gIC receive orofacial nociceptive signals originating from the periodontal ligament (PDL) and dental pulp [78].

### Neurofunctional dynamics of the insular cortex in trigeminal neuropathic pain processing pathway

The IC is a key component of the pain matrix, which is involved in the multidimensional aspects of pain perception, including the SI and SII, anterior cingulate cortex, PFC, thalamus, and cerebellar cortices. It is consistently activated during TNP and plays a pivotal role in both the sensory-discriminative and affective-motivational dimensions of TNP, mediating bottom-up and top-down modulation [3, 10, 57]. It influences both antinociceptive and pronociceptive pathways through reciprocal glutamatergic projections to essential brain areas such as the somatosensory, motor, and prefrontal cortices, as well as the striatum, amygdala, and thalamus [38, 43, 69, 101].

After trigeminal nerve injury, nociceptive signals are transmitted from second-order neurons in the TNC to the VPM thalamus and medial nucleus of the posterior complex, which maintain bidirectional connections with the IC. Hence, innocuous mechanical stimuli can activate the nociceptive-specific neurons situated in the layers II-III of IC to influence TNP states [3, 43]. On the other hand, direct descending projections from the IC to the TNC suggest its facilitatory role in TNP. Post-injury, trigeminal nerve projections activate nociceptive neurons in the outer (I-II) and inner (V-VI) laminae of the spinal and medullary dorsal horn, and dIC in rodents has been observed to send crucial contralateral projections to these areas and the brainstem, modulating orofacial nociceptive processing [2, 56, 69, 84, 89].

In the IC, 73% of neurons are excitatory glutamatergic and 27% are inhibitory GABAergic. The interaction of IC with NMDA receptors influences antinociceptive effects through descending pain modulatory pathways. Post trigeminal nerve injury, changes in AMPA receptor composition in the IC enhance synaptic Ca2 + permeability, which also facilitates long-term potentiation (LTP) through increased glutamatergic transmission [52, 84, 98]. Additionally, the serotoninergic system in the IC significantly influences chronic pain mechanisms, with 5-HT and 5-HIAA levels rising after trigeminal nerve injuries. Over 70% of glutamatergic neurons in the IC express 5-HT1A receptors, indicating a broad serotonergic influence on its neurochemical circuits [17, 52, 84]. Therefore, neurochemical imbalances in the IC facilitated by increased glutamatergic activity, evidenced by elevated c-Fos expression in these neurons post-CCI-ION surgery, highlight its role in TNP and central sensitization. This neurochemical imbalance is evident in both animal models and human studies of TNP [50, 58, 77, 102].

### Neuromodulation approaches involving the insular cortex in trigeminal neuropathic pain management

**Fig. 2:**
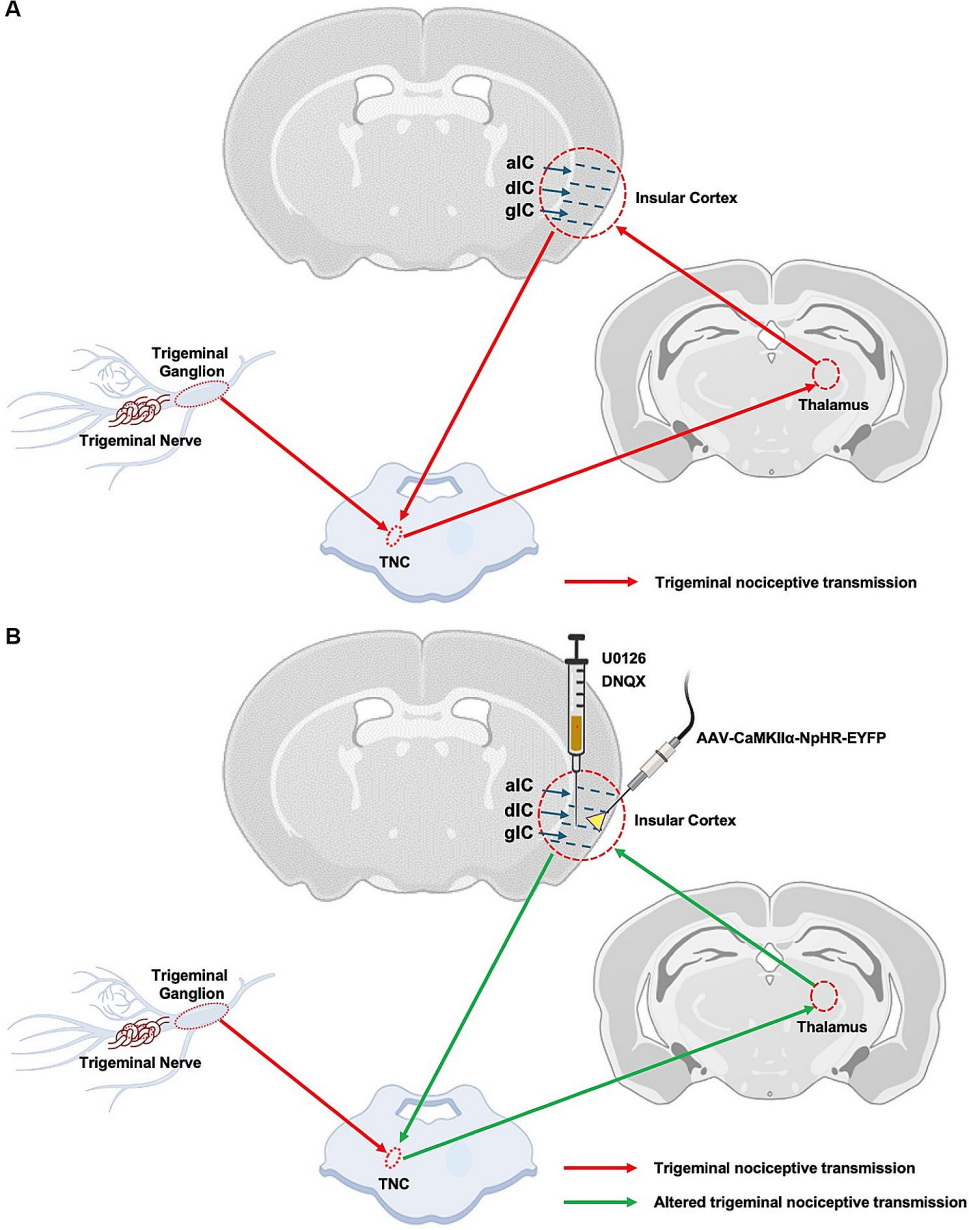
Modulation of IC activity alters trigeminal pain processing pathway. **(A)** Trigeminal nociceptive transmission pathway via IC after trigeminal nerve injury. **(B)** Altered trigeminal nociceptive transmission pathway after IC neuromodulation to improve TNP condition. U0126 = ERK inhibitor, DNQX = AMPA receptor blocker, AAV-CaMKIIα-NpHR-EYFP = optogenetic virus targeting glutamatergic neurons, gIC = granular insular cortex, dIC = dysgranular insular cortex, aIC = agranular insular cortex, TNC = trigeminal nucleus caudalis

The IC holds significant therapeutic potential for managing TNP. Although relatively few, several preclinical studies employing chemical interventions and neuromodulation techniques, such as optogenetics, have shown that modulating the activity of IC plays a crucial role in altering TNP processing. These findings suggest that interventions targeting IC could significantly modify pain perception and emotional responses to TNP (Fig. 2). Hence, it is imperative to conduct extensive studies in both clinical and preclinical domains for the development of personalized medicine approaches that consider the individual variability in the structure and function of the IC as it could lead to effective and tailored treatments for those suffering from TNP.

### Chemical modulation

Wang et al. [98], demonstrated that corticotrigeminal projections from the IC to the TNC regulate orofacial pain and negative emotions in CCI-ION rats. This regulation occurs through the activation of the ERK pathway in IC (mainly in gIC/dIC) neurons. Infusion of U0126, an inhibitor of ERK activation, was shown to decrease both the upregulation of p-ERK in the IC and the expression of Fos in the TNC, thereby alleviating nociceptive behaviors and negative emotions in rats with nerve injury. This suggests that deactivating IC neurons by inhibiting ERK phosphorylation could significantly lessen orofacial neuropathic pain caused by CCI-ION [98].

Administration of the AMPA receptor blocker DNQX within the IC was found to decrease excitatory postsynaptic potential (EPSP) activity in the spinal trigeminal complex (Sp5C), indicating the role of AMPA receptors in the IC in the transmission of nociceptive signals [77].

### Optogenetic modulation

In our recent study, we investigated the effect of optogenetic modulation of dPICg on TNP in a CCI-ION rat model. We found that optogenetic inhibition of dPICg decreased neural firing rates in the TNC and the VPM thalamus, reduced expression of sensory-responsive cell bodies and transcriptional factors in the dPIC, and improved hyperalgesia, allodynia, and anxiety-like responses in CCI-ION animals. This highlights the potential antinociceptive value of precisely inhibiting certain neural populations within the IC for managing TNP [50].

### Potential prospects for insular cotrex modulation in clinical studies

Transcranial direct current stimulation (tDCS) is a non-invasive brain stimulation technique that has shown promising results in inducing lasting changes in brain activity. Although tDCS predominantly influences more superficial brain regions, high-definition montages (HD-tDCS) has proven effective in modulating PIC activity. Consequently, cathodal HD-tDCS targeting the PIC could offer new avenues for TNP management [41]. On the other hand, indirect modulation of IC through tDCS applied to other cortical areas that have direct reciprocal connections with IC may also hold therapeutic potential. For instance, anodal tDCS applied to the motor cortex has demonstrated a reduction in CNP intensity among patients with multiple sclerosis [74]. In addition, cathodal tDCS applied to SSC could modulate PIC activity [55]. Future advancements, including focused stimulation techniques and enhanced electrode designs, and continued research on the application of tDCS for IC modulation in TNP are necessary to fully ascertain the potential of tDCS in clinical settings for effective TNP management.

Transcranial Magnetic Stimulation (TMS) also offers a promising approach to modulating the IC for managing TNP. TMS utilizes magnetic fields to induce electrical currents in specific brain areas, and with advancements such as deep TMS (dTMS), it can target deeper structures like the IC [65, 101]. Clinical evidence shows that TMS applied to the PIC in patients with central neuropathic pain can increase thermal thresholds, indicating a modulation of pain sensitivity, though without a significant effect on neuropathic pain scores. Conversely, in peripheral neuropathic pain patients, repeated TMS sessions have demonstrated a significant, albeit short-lasting, analgesic effect [61]. These findings suggest that while the direct modulation of the IC using TMS presents certain challenges due to the depth of the target area, innovative approaches in TMS technology could enhance its efficacy in modulating insular activity, thus offering a non-pharmacological therapeutic option for TNP management. Continued research into TMS application specifics, such as stimulation parameters and session frequency, will be critical in optimizing its use for effective TNP relief in clinical settings.

## Discussion

IC in TNP management warrants specific consideration due to its unique anatomical connections, notably the direct pathways between the trigeminal nerve and the IC. This specialized interaction is critical for understanding the intense and debilitating nature of TNP. During TNP, the IC exhibits distinct activation patterns and neuroplastic changes such as decreased GMV in the AIC, increased GMV in the PIC, increased glutamatergic neurotransmissions, reflecting long-term adaptations to chronic pain [101]; Wang et al. [50, 61, 68, 100]. Consequently, IC neuromodulation holds significant potential for managing TNP, offering a targeted approach to modulate these complex pain pathways effectively.

Advanced neuroimaging techniques, including PET and magnetic resonance imaging (MRI), play crucial part for identifying neuroplastic changes within the IC, serving as potential biomarkers for TNP [23, 24, 101]. These tools help differentiate TNP from other facial pains, enabling more precise and timely interventions through the integration of imaging results with clinical evaluations. Pharmacological manipulation with selective serotonin reuptake inhibitors (SSRIs) and serotonin noradrenaline reuptake inhibitors (SNRIs) could have efficacy in modifying the affective components of orofacial pain since the IC serotoninergic system significantly influences the chronicity of pain [13, 17]. Recent advancements in neuromodulation, such as transcranial magnetic stimulation (TMS) and deep brain stimulation (DBS), have also shown promise in targeting the IC to alleviate TNP symptoms by reshaping pain processing. In addition, the introduction of optogenetic stimulation provides a precise method to control the neuronal activity in IC, furthering research in trigeminal pain pathways [12, 50, 106].

The existing gaps in research regarding the IC’s role in TNP primarily stem from a scarcity of direct comparative studies between TNP and other neuropathies. Such comparative studies are essential to identify unique neurobiological and pathophysiological features of TNP, especially those mediated by the IC. Understanding these unique features will facilitate developing specific therapeutic strategies that are finely tuned to the nuances of TNP, potentially leading to more effective treatments. Moreover, there is a notable deficiency in longitudinal research that tracks the progression of IC involvement from the acute phase of TNP to its chronic state. Most existing studies provide only cross-sectional data, capturing a single moment in the disease’s progression. Longitudinal studies would allow researchers to observe how the role of the IC evolves over time, offering insights into the development of chronic TNP.

Therefore, looking ahead, the integration of neuromodulation, advanced diagnostics, and pharmacological strategies involving IC is crucial for a holistic approach to TNP management. Longitudinal studies and genetic research are essential to assess the efficacy of IC-targeted therapies and tailor treatments to individual responses. Combining neuromodulation techniques such as TMS, DBS, and optogenetic or chemogenetic methods could reveal new TNP relief mechanisms. Cross-disciplinary collaborations are also essential for increasing awareness among healthcare professionals and patients about the IC’s role in TNP which can enhance disease recognition and encourage exploration of new treatments.

## Conclusion

This review compiles research on the critical role of IC subdivisions in TNP. TNP is associated with IC dysfunction, characterized by early changes in glutamatergic receptor plasticity that lead to pain chronification, maladaptive pERK signaling, and disruptions in GABAergic and dopaminergic systems. Understanding the IC’s influence on TNP requires further preclinical studies that build on clinical insights. This review will contribute to the new perspectives on directly targeting the IC for the effective TNP management.

## Data Availability

No datasets were generated or analysed during the current study.

## Abbreviations

IC: Insular cortex
aIC: Agranular insular cortex
dIC: Dysgranular insular cortex
gIC: Granular insular cortex
AIC: Anterior insular cortex
PIC: Posterior insular cortex
dPICg: Glutamatergic neurons of dysgranular posterior insular cortex
TNP: Trigeminal neuropathic pain
TN: Trigeminal neuralgia
CCI-ION: Chronic constriction of infraorbital nerve
TNC: Trigeminal nucleus caudalis
GMV: Gray matter volume
WMV: White matter volume
pERK: Phospho-Extracellular Signal-Regulated Kinase
fMRI: Functional Magnetic Resonance Imaging
DWI: Diffusion-weighted magnetic resonance imaging
SVM: Support vector machine classification
VBM: Voxel-based morphometry
CTA: Cortical thickness analysis
OFC: Orbitofrontal cortex
PFC: Prefrontal cortex
MC: Motor cortex
SSC: Somatosensory cortex
SI: Primary somatosensory cortices
SII: Secondary somatosensory cortices
NAc: Nucleus accumbens
NAcC: Nucleus accumbens core
VPM: Ventral posteromedial
VPL: Ventral posterolateral
CeA: Central amygdala
PBN: Parabrachial nucleus
LC: Locus coeruleus
RVM: Rostral ventromedial medulla
PAG: Periaqueductal gray
LH: Lateral hypothalamus

## References

[1] Abdallah M, Khalil S, Hamed A, Soliman RK, Mohamed A (2020). Brain activity assessment by functional MRI before and after radiofrequency of gasserian ganglia in patients with trigeminal neuralgia. Anaesth Pain Intensive Care.

[2] Akhter F, Haque T, Sato F, Kato T, Ohara H, Fujio T, Tsutsumi K, Uchino K, Sessle B, Yoshida A (2014). Projections from the dorsal peduncular cortex to the trigeminal subnucleus caudalis (medullary dorsal horn) and other lower brainstem areas in rats. Neuroscience.

[3] Alvarez P, Dieb W, Hafidi A, Voisin DL, Dallel R (2009). Insular cortex representation of dynamic mechanical allodynia in trigeminal neuropathic rats. Neurobiol Dis.

[4] Araya EI, Carvalho EC, Andreatini R, Zamponi GW, Chichorro JG (2022). Trigeminal neuropathic pain causes changes in affective processing of pain in rats. Mol Pain.

[5] Avery JA, Drevets WC, Moseman SE, Bodurka J, Barcalow JC, Simmons WK (2014). Major depressive disorder is associated with abnormal interoceptive activity and functional connectivity in the insula. Biol Psychiatry.

[6] Barrett LF, Simmons WK (2015). Interoceptive predictions in the brain. Nat Rev Neurosci.

[7] Baumgärtner U, Iannetti GD, Zambreanu L, Stoeter P, Treede R-D, Tracey I (2010). Multiple somatotopic representations of heat and mechanical pain in the operculo-insular cortex: a high-resolution fMRI study. J Neurophysiol.

[8] Becerra L, Morris S, Bazes S, Gostic R, Sherman S, Gostic J, Pendse G, Moulton E, Scrivani S, Keith D (2006). Trigeminal neuropathic pain alters responses in CNS circuits to mechanical (brush) and thermal (cold and heat) stimuli. J Neurosci.

[9] Bendtsen L, Zakrzewska JM, Heinskou TB, Hodaie M, Leal PRL, Nurmikko T, Obermann M, Cruccu G, Maarbjerg S (2020). Advances in diagnosis, classification, pathophysiology, and management of trigeminal neuralgia. Lancet Neurol.

[10] Benison AM (2012) Insular cortex: functional mapping and allodynic behavior in the rat. University of Colorado at Boulder

[11] Benison AM, Chumachenko S, Harrison JA, Maier SF, Falci SP, Watkins LR, Barth DS (2011). Caudal granular insular cortex is sufficient and necessary for the long-term maintenance of allodynic behavior in the rat attributable to mononeuropathy. J Neurosci.

[12] Bergeron D, Obaid S, Fournier-Gosselin M-P, Bouthillier A, Nguyen DK (2021). Deep brain stimulation of the posterior insula in chronic pain: a theoretical framework. Brain Sci.

[13] Bonilla-Jaime H, Sánchez-Salcedo JA, Estevez-Cabrera MM, Molina-Jiménez T, Cortes-Altamirano JL, Alfaro-Rodríguez A (2022). Depression and pain: use of antidepressants. Curr Neuropharmacol.

[14] Chen X, Gabitto M, Peng Y, Ryba NJ, Zuker CS (2011). A gustotopic map of taste qualities in the mammalian brain. Science.

[15] Coffeen U, López-Avila A, Ortega-Legaspi JM, Del Ángel R, López-Muñoz FJ, Pellicer F (2008). Dopamine receptors in the anterior insular cortex modulate long-term nociception in the rat. Eur J Pain.

[16] Coffeen U, Ortega-Legaspi JM, López-Muñoz FJ, Simón-Arceo K, Jaimes O, Pellicer F (2011). Insular cortex lesion diminishes neuropathic and inflammatory pain-like behaviours. Eur J Pain.

[17] Coffeen U, Canseco-Alba A, Simón-Arceo K, Mercado F, Almanza A, Jaimes O, Pellicer F (2016). Extracellular levels of 5HT and 5HIAA increase after an inflammatory process in the rat’s insular cortex. World J Neurosci.

[18] Craig A (2004). Distribution of trigeminothalamic and spinothalamic lamina I terminations in the macaque monkey. J Comp Neurol.

[19] Craig AD (2009). How do you feel—now? The anterior insula and human awareness. Nat Rev Neurosci.

[20] Craig AD, Chen K, Bandy D, Reiman EM (2000). Thermosensory activation of insular cortex. Nat Neurosci.

[21] Critchley HD, Harrison NA (2013). Visceral influences on brain and behavior. Neuron.

[22] Cruccu G, Di Stefano G, Truini A (2020). Trigeminal neuralgia. N Engl J Med.

[23] DaSilva AF, Becerra L, Pendse G, Chizh B, Tully S, Borsook D (2008). Colocalized structural and functional changes in the cortex of patients with trigeminal neuropathic pain. PLoS ONE.

[24] DaSilva AF, Zubieta J-K, DosSantos MF (2019). Positron emission tomography imaging of endogenous mu-opioid mechanisms during pain and migraine. Pain Rep.

[25] Desbois C, Le Bars D, Villanueva L (1999). Organization of cortical projections to the medullary subnucleus reticularis dorsalis: a retrograde and anterograde tracing study in the rat. J Comp Neurol.

[26] DeSouza DD, Moayedi M, Chen DQ, Davis KD, Hodaie M (2013). Sensorimotor and pain modulation brain abnormalities in trigeminal neuralgia: a paroxysmal, sensory-triggered neuropathic pain. PLoS ONE.

[27] DeSouza DD, Davis KD, Hodaie M (2015). Reversal of insular and microstructural nerve abnormalities following effective surgical treatment for trigeminal neuralgia. Pain.

[28] Devue C, Collette F, Balteau E, Degueldre C, Luxen A, Maquet P, Brédart S (2007). Here I am: the cortical correlates of visual self-recognition. Brain Res.

[29] Donertas-Ayaz B, Caudle RM (2023). Locus coeruleus-noradrenergic modulation of trigeminal pain: implications for trigeminal neuralgia and psychiatric comorbidities. Neurobiol Pain.

[30] Droutman V, Read SJ, Bechara A (2015). Revisiting the role of the insula in addiction. Trends Cogn Sci.

[31] Emmert K, Breimhorst M, Bauermann T, Birklein F, Van De Ville D, Haller S (2014). Comparison of anterior cingulate vs. insular cortex as targets for real-time fMRI regulation during pain stimulation. Front Behav Neurosci.

[32] Etkin A, Wager TD (2007). Functional neuroimaging of anxiety: a meta-analysis of emotional processing in PTSD, social anxiety disorder, and specific phobia. Am J Psychiatry.

[33] Evrard HC (2019). The organization of the primate insular cortex. Front Neuroanat.

[34] Fermin AS, Friston K, Yamawaki S (2021) Insula interoception, active inference and feeling representation. arXiv Preprint. arXiv:211212290

[35] Fujita S, Yamamoto K, Kobayashi M (2019) Trigeminal nerve transection-induced neuroplastic changes in the somatosensory and insular cortices in a rat ectopic pain model. Eneuro 6 (1) 10.1523/ENEURO.0462-18.2019 PMC6348450 30693315

[36] Gallay DS, Gallay M, Jeanmonod D, Rouiller EM, Morel A (2012). The insula of Reil revisited: multiarchitectonic organization in macaque monkeys. Cereb Cortex.

[37] Gambeta E, Chichorro JG, Zamponi GW (2020). Trigeminal neuralgia: an overview from pathophysiology to pharmacological treatments. Mol Pain.

[38] Gehrlach DA, Dolensek N, Klein AS, Roy Chowdhury R, Matthys A, Junghänel M, Gaitanos TN, Podgornik A, Black TD, Reddy Vaka N (2019). Aversive state processing in the posterior insular cortex. Nat Neurosci.

[39] Gehrlach DA, Weiand C, Gaitanos TN, Cho E, Klein AS, Hennrich AA, Conzelmann K-K, Gogolla N (2020). A whole-brain connectivity map of mouse insular cortex. Elife.

[40] Gogolla N (2017). The insular cortex. Curr Biol.

[41] Gorrino I, Canessa N, Mattavelli G (2023) Testing the effect of high-definition transcranial direct current stimulation of the insular cortex to modulate decision-making and executive control. Front Behav Neurosci 17 10.3389/fnbeh.2023.1234837 PMC10568024 37840546

[42] Gustin SM, Peck CC, Wilcox SL, Nash PG, Murray GM, Henderson LA (2011). Different pain, different brain: thalamic anatomy in neuropathic and non-neuropathic chronic pain syndromes. J Neurosci.

[43] Gutzeit A, Meier D, Meier M, von Weymarn C, Ettlin DA, Graf N, Froehlich J, Binkert C, Brügger M (2011). Insula-specific responses induced by dental pain. A proton magnetic resonance spectroscopy study. Eur Radiol.

[44] Han J, Kwon M, Cha M, Tanioka M, Hong S-K, Bai SJ, Lee BH (2015) Plasticity-related PKMζ signaling in the insular cortex is involved in the modulation of neuropathic pain after nerve injury. Neural plasticity 2015 10.1155/2015/601767 PMC4592717 26457205

[45] Hassanzadeh R, Jones JC, Ross EL (2014). Neuromodulation for intractable headaches. Curr Pain Headache Rep.

[46] Hayes DJ, Chen DQ, Zhong J, Lin A, Behan B, Walker M, Hodaie M (2017) Affective circuitry alterations in patients with trigeminal neuralgia. Front Neuroanat:73 10.3389/fnana.2017.00073 PMC5591854 28928638

[47] Henssen D, Dijk J, Knepflé R, Sieffers M, Winter A, Vissers K (2019). Alterations in grey matter density and functional connectivity in trigeminal neuropathic pain and trigeminal neuralgia: a systematic review and meta-analysis. NeuroImage: Clin.

[48] Iezzi D, Cáceres-Rodríguez A, Strauss B, Chavis P, Manzoni OJ (2024). Sexual differences in neuronal and synaptic properties across subregions of the mouse insular cortex. Biology sex Differences.

[49] Islam J, Kc E, Kim S, Kim HK, Park YS (2021). Stimulating gabaergic neurons in the nucleus accumbens core alters the trigeminal neuropathic pain responses in a rat model of infraorbital nerve injury. Int J Mol Sci.

[50] Islam J, Kc E, Kim S, Chung MY, Park KS, Kim HK, Park YS (2023). Optogenetic Inhibition of Glutamatergic Neurons in the dysgranular posterior insular cortex modulates Trigeminal Neuropathic Pain in CCI-ION rat. Neuromol Med.

[51] Jasmin L, Rabkin SD, Granato A, Boudah A, Ohara PT (2003). Analgesia and hyperalgesia from GABA-mediated modulation of the cerebral cortex. Nature.

[52] Ju A, Fernandez-Arroyo B, Wu Y, Jacky D, Beyeler A (2020). Expression of serotonin 1A and 2A receptors in molecular-and projection-defined neurons of the mouse insular cortex. Mol Brain.

[53] Kargl D, Kaczanowska J, Ulonska S, Groessl F, Piszczek L, Lazovic J, Buehler K, Haubensak W (2020). The amygdala instructs insular feedback for affective learning. Elife.

[54] Kaushal R, Taylor BK, Jamal A, Zhang L, Ma F, Donahue R, Westlund K (2016). GABA-A receptor activity in the noradrenergic locus coeruleus drives trigeminal neuropathic pain in the rat; contribution of NAα1 receptors in the medial prefrontal cortex. Neuroscience.

[55] Knotkova H, Nitsche MA, Cruciani RA (2013). Putative physiological mechanisms underlying tDCS analgesic effects. Front Hum Neurosci.

[56] Kobayashi M (2011). Macroscopic connection of rat insular cortex: anatomical bases underlying its physiological functions. Int Rev Neurobiol.

[57] Kobayashi M (2018). Mechanisms of orofacial sensory processing in the rat insular cortex. J Oral Biosci.

[58] Kobayashi M, Nakaya Y (2020). Anatomical aspects of corticotrigeminal projections to the medullary dorsal horn. J Oral Sci.

[59] Koga K, Li S, Zhuo M (2016). Metabotropic glutamate receptor dependent cortical plasticity in chronic pain. Curr Neuropharmacol.

[60] Krushel LA, van Der Kooy D (1988). Visceral cortex: integration of the mucosal senses with limbic information in the rat agranular insular cortex. J Comp Neurol.

[61] Labrakakis C (2023). The role of the insular cortex in Pain. Int J Mol Sci.

[62] Lau T, Schloss P (2008). The cannabinoid CB1 receptor is expressed on serotonergic and dopaminergic neurons. Eur J Pharmacol.

[63] Lee C-H, Jang H-Y, Won H-S, Kim J-S, Kim Y-D (2021). Epidemiology of trigeminal neuralgia: an electronic population health data study in Korea. Korean J pain.

[64] Lin C-s (2014). Brain signature of chronic orofacial pain: a systematic review and meta-analysis on neuroimaging research of trigeminal neuropathic pain and temporomandibular joint disorders. PLoS ONE.

[65] Lindholm P (2017) Neural mechanisms of orofacial pain-effects of transcranial magnetic stimulation

[66] Liu Y, Chen Q-Y, Lee JH, Li X-H, Yu S, Zhuo M (2020). Cortical potentiation induced by calcitonin gene-related peptide (CGRP) in the insular cortex of adult mice. Mol Brain.

[67] Livneh Y, Sugden AU, Madara JC, Essner RA, Flores VI, Sugden LA, Resch JM, Lowell BB, Andermann ML (2020). Estimation of current and future physiological states in insular cortex. Neuron.

[68] Lötsch J, Walter C, Felden L, Nöth U, Deichmann R, Oertel BG (2012). The human operculo-insular cortex is pain-preferentially but not pain-exclusively activated by trigeminal and olfactory stimuli. PLoS ONE.

[69] Lu C, Yang T, Zhao H, Zhang M, Meng F, Fu H, Xie Y, Xu H (2016). Insular cortex is critical for the perception, modulation, and chronification of pain. Neurosci Bull.

[70] Mathiasen ML, Aggleton JP, Witter MP (2023). Projections of the insular cortex to orbitofrontal and medial prefrontal cortex: a tracing study in the rat. Front Neuroanat.

[71] Mazzola L, Isnard J, Peyron R, Guénot M, Mauguiere F (2009). Somatotopic organization of pain responses to direct electrical stimulation of the human insular cortex. Pain.

[72] Moisset X, Villain N, Ducreux D, Serrie A, Cunin G, Valade D, Calvino B, Bouhassira D (2011). Functional brain imaging of trigeminal neuralgia. Eur J Pain.

[73] Montano N, Conforti G, Di Bonaventura R, Meglio M, Fernandez E, Papacci F (2015) Advances in diagnosis and treatment of trigeminal neuralgia. Therapeutics and clinical risk management:289–299 10.2147/TCRM.S37592 PMC4348120 25750533

[74] Mori F, Codecà C, Kusayanagi H, Monteleone F, Buttari F, Fiore S, Bernardi G, Koch G, Centonze D (2010). Effects of anodal transcranial direct current stimulation on chronic neuropathic pain in patients with multiple sclerosis. J pain.

[75] Muñoz-Castañeda R, Zingg B, Matho KS, Chen X, Wang Q, Foster NN, Li A, Narasimhan A, Hirokawa KE, Huo B (2021). Cellular anatomy of the mouse primary motor cortex. Nature.

[76] Mutschler I, Hänggi J, Frei M, Lieb R, Grosse Holforth M, Seifritz E, Spinelli S (2019). Insular volume reductions in patients with major depressive disorder. Neurol Psychiatry Brain Res.

[77] Nakaya N, Yamamoto Y, Kobayashi K (2022) Descending projections from the insular cortex to the trigeminal spinal subnucleus caudalis facilitate excitatory outputs to the parabrachial nucleus in rats. Pain. 10.1097 10.1097/j.pain.0000000000002755 PMC9916064 35969237

[78] Nakaya Y, Iwata K, Kobayashi M (2023). Insular cortical descending projections facilitate neuronal responses to noxious but not innoxious stimulation in rat trigeminal spinal subnucleus caudalis. Brain Res.

[79] Namkung H, Kim S-H, Sawa A (2017). The insula: an underestimated brain area in clinical neuroscience, psychiatry, and neurology. Trends Neurosci.

[80] Noma D, Fujita S, Zama M, Mayahara K, Motoyoshi M, Kobayashi M (2020). Application of oxytocin with low-level laser irradiation suppresses the facilitation of cortical excitability by partial ligation of the infraorbital nerve in rats: an optical imaging study. Brain Res.

[81] Noseda R, Constandil L, Bourgeais L, Chalus M, Villanueva L (2010). Changes of meningeal excitability mediated by corticotrigeminal networks: a link for the endogenous modulation of migraine pain. J Neurosci.

[82] Paulus MP, Stein MB (2006). An insular view of anxiety. Biol Psychiatry.

[83] Peltz E, Seifert F, DeCol R, Dörfler A, Schwab S, Maihöfner C (2011). Functional connectivity of the human insular cortex during noxious and innocuous thermal stimulation. NeuroImage.

[84] Pereira RCM, Medeiros P, Coimbra NC, Machado HR, de Freitas RL (2023). Cortical neurostimulation and N-methyl-D-aspartate glutamatergic receptor activation in the dysgranular layer of the posterior insular cortex modulate chronic neuropathic pain. Neuromodulation: Technol Neural Interface.

[85] Ramos-Prats A, Paradiso E, Castaldi F, Sadeghi M, Mir MY, Hörtnagl H, Göbel G, Ferraguti F (2022) VIP-expressing interneurons in the anterior insular cortex contribute to sensory processing to regulate adaptive behavior. Cell Rep 39 (9) 10.1016/j.celrep.2022.110893 35649348

[86] Rodgers KM, Benison AM, Klein A, Barth DS (2008). Auditory, somatosensory, and multisensory insular cortex in the rat. Cereb Cortex.

[87] Rousseau C, Barbiero M, Pozzo T, Papaxanthis C, White O (2021). Actual and imagined movements reveal a dual role of the insular cortex for motor control. Cereb Cortex.

[88] Saper CB (2007) Visceral sensation and visceral sensory disorders. CONTINUUM: Lifelong Learn Neurol 13(6):204–214

[89] Sato F, Akhter F, Haque T, Kato T, Takeda R, Nagase Y, Sessle B, Yoshida A (2013). Projections from the insular cortex to pain-receptive trigeminal caudal subnucleus (medullary dorsal horn) and other lower brainstem areas in rats. Neuroscience.

[90] Shi CJ, Cassell M (1998). Cortical, thalamic, and amygdaloid connections of the anterior and posterior insular cortices. J Comp Neurol.

[91] Silva M, Ouanounou A (2020). Trigeminal neuralgia: etiology, diagnosis, and treatment. SN Compr Clin Med.

[92] Taylor KS, Seminowicz DA, Davis KD (2009). Two systems of resting state connectivity between the insula and cingulate cortex. Hum Brain Mapp.

[93] Tsagareli N, Tsiklauri N, Kvachadze I, Tsagareli MG (2020). Endogenous opioid and cannabinoid systems contribute to antinociception produced by administration of NSAIDs into the insular cortex of rats. Biomed Pharmacother.

[94] Uddin LQ (2015). Salience processing and insular cortical function and dysfunction. Nat Rev Neurosci.

[95] Uddin LQ, Menon V (2009). The anterior insula in autism: under-connected and under-examined. Neurosci Biobehavioral Reviews.

[96] Unger N, Haeck M, Eickhoff SB, Camilleri JA, Dickscheid T, Mohlberg H, Bludau S, Caspers S, Amunts K (2023) Cytoarchitectonic mapping of the human frontal operculum—new correlates for a variety of brain functions. Front Hum Neurosci 17 10.3389/fnhum.2023.1087026 PMC10336231 37448625

[97] Veinante P, Yalcin I, Barrot M (2013). The amygdala between sensation and affect: a role in pain. J Mol Psychiatry.

[98] Wang J, Li Z-H, Feng B, Zhang T, Zhang H, Li H, Chen T, Cui J, Zang W-D, Li Y-Q (2015). Corticotrigeminal projections from the insular cortex to the trigeminal caudal subnucleus regulate orofacial pain after nerve injury via extracellular signal-regulated kinase activation in insular cortex neurons. Front Cell Neurosci.

[99] Wang Y, Cao D-y, Remeniuk B, Krimmel S, Seminowicz DA, Zhang M (2017). Altered brain structure and function associated with sensory and affective components of classic trigeminal neuralgia. Pain.

[100] Wang Y, Zhang Y, Zhang J, Wang J, Xu J, Li J, Cui G, Zhang J (2018). Structural and functional abnormalities of the insular cortex in trigeminal neuralgia: a multimodal magnetic resonance imaging analysis. Pain.

[101] Wang N, Zhang Y-H, Wang J-Y, Luo F (2021). Current understanding of the involvement of the insular cortex in neuropathic pain: a narrative review. Int J Mol Sci.

[102] Watson CJ (2016). Insular balance of glutamatergic and GABAergic signaling modulates pain processing. Pain.

[103] Xu R, Zhang YW, Gu Q, Yuan TJ, Fan BQ, Xia JM, Wu JH, Xia Y, Li WX, Han Y (2023) Brain function activity changes and contribution of neuroinflammatory factors in Insular Cortex of mice. with Dry Eye-Related Chronic Corneal Pain 10.1111/gbb.12842 PMC10067426 36889983

[104] Yasui Y, Breder CD, Safer CB, Cechetto DF (1991). Autonomic responses and efferent pathways from the insular cortex in the rat. J Comp Neurol.

[105] Zhang Y, Mao Z, Pan L, Ling Z, Liu X, Zhang J, Yu X (2018). Dysregulation of pain-and emotion-related networks in trigeminal neuralgia. Front Hum Neurosci.

[106] Zhang K-L, Yuan H, Wu F-F, Pu X-Y, Liu B-Z, Li Z, Li K-F, Liu H, Yang Y, Wang Y-Y (2021). Analgesic effect of noninvasive brain stimulation for neuropathic pain patients: a systematic review. Pain Therapy.

[107] Zhong J, Chen DQ, Hung PS-P, Hayes DJ, Liang KE, Davis KD, Hodaie M (2018). Multivariate pattern classification of brain white matter connectivity predicts classic trigeminal neuralgia. Pain.

